# 18-Fluorodeoxyglucose Positron Emission Tomography/Computed Tomography in the Management of Aggressive Non-Hodgkin's B-Cell Lymphoma

**DOI:** 10.5402/2012/456706

**Published:** 2012-03-11

**Authors:** M. J. Shelly, S. McDermott, O. J. O'Connor, M. A. Blake

**Affiliations:** ^1^Department of Radiology, Massachusetts General Hospital, Boston, MA 02114, USA; ^2^Division of Abdominal Imaging & Interventional Radiology, Massachusetts General Hospital, Harvard Medical School, Boston, MA 02114, USA

## Abstract

18-Fluorodeoxyglucose (FDG-PET/CT) is an established imaging modality that has been proven to be of benefit in the management of aggressive B-cell non-Hodgkin's lymphoma, such as diffuse large B-cell lymphoma and advanced stage follicular lymphoma. The combination of anatomic and functional imaging afforded by FDG-PET/CT has led to superior sensitivity and specificity in the primary staging, restaging, and assessment of response to treatment of hematological malignancies when compared to FDG-PET and CT alone. The use of FDG-PET/CT for posttreatment surveillance imaging remains controversial, and further study is needed to ascertain whether this modality is cost effective and appropriate for use in this setting.

## 1. Introduction

The lymphoproliferative disorders, which can be broadly divided into Hodgkin's (HL) and non-Hodgkin's (NHL) lymphoma, represent a heterogeneous group of lymphoid malignancies that display varying patterns of biological behavior and response to treatment [1]. It is estimated that over 65,000 Americans will be diagnosed with NHL with over 20,000 deaths in 2011, making it the eighth and sixth most common cause of cancer death in men and women, respectively [2]. The annual incidence of NHL is trending upwards year on year; however, the overall 5-year survival rate has been steadily improving due in part to refinements in treatment regimens which minimize patient toxicity and maximize remission rates. Classification of lymphomas according to the 2008 World Health Organization classification system groups lymphomas by cell type (i.e., the normal cell type that most resembles the tumor) and defines phenotypic, molecular, or cytogenetic characteristics with three major groupings, namely, the B-cell, T-cell, and natural killer cell neoplasms [3]. The most common subtypes of NHL affecting adults are follicular lymphoma (FL) and diffuse large B-cell lymphoma (DLBCL), which together account for greater than 50% of NHL [4, 5]. Prognosis of patients with NHL is affected by the stage and grade of disease as well as the histological subtype. Physicians often classify NHL into indolent, aggressive, and highly aggressive histologic subtypes for the purpose of estimating prognosis and determining treatment options. This paper will discuss the role of FDG-PET/CT in the pretreatment staging, restaging, treatment monitoring, assessment of transformation, and posttherapy surveillance of patients with aggressive non-Hodgkin's lymphoma of B-cell origin. The discussion of the role of FDG-PET/CT in the management of Hodgkin's disease is beyond the scope of this paper.

## 2. Imaging Considerations in Lymphoma

**Figure 1:**
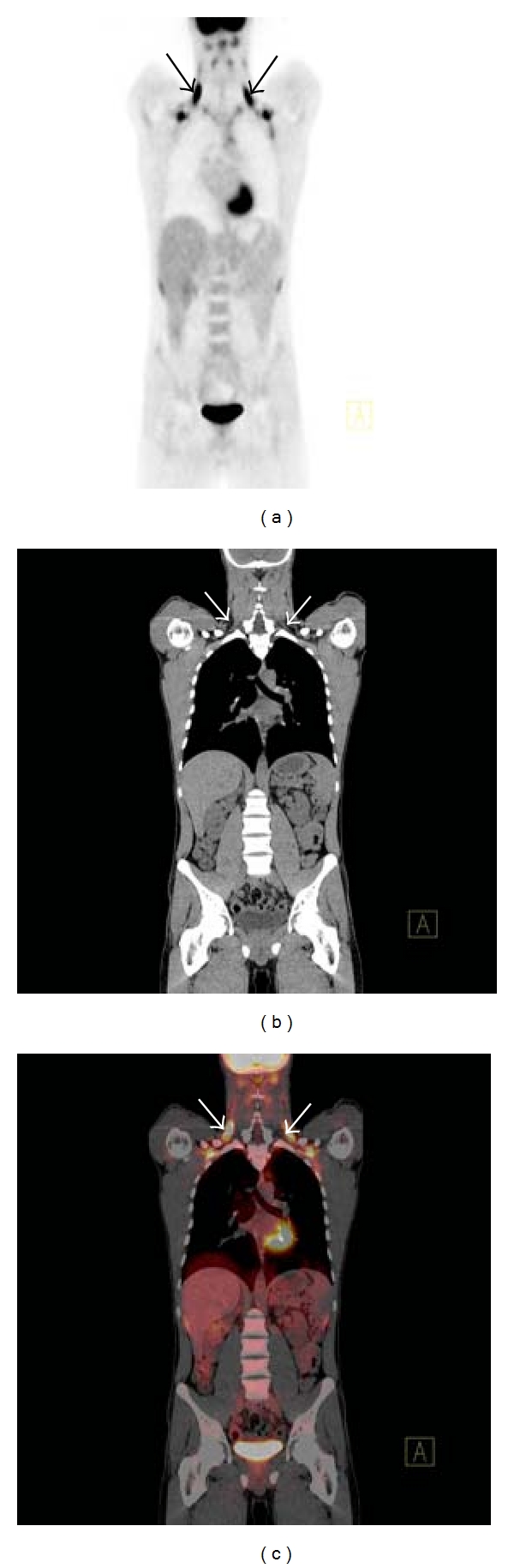
Coronal PET maximum intensity projection (MIP) image (a) displaying foci of increased radiotracer uptake bilaterally in the neck in a patient with treated DLBSL. The corresponding coronal CT (b) and fused FDG-PET/CT (c) demonstrate no abnormality and the areas with increased FDG uptake correspond to regions of fat density (arrows). This is the characteristic appearance of hypermetabolic brown fat on FDG-PET/CT.

The management of lymphoma depends on histologic subtype, stage, and grade. HL and the more aggressive forms of NHL share similar radiologic imaging patterns with the near universal use of combined FDG-PET/CT for assessment [6, 7]. Combined FDG-PET/CT is now considered the most accurate tool for diagnosis, assessment of treatment response, and prognosis in patients with aggressive NHL [8]. There are a number of caveats that one must be aware of when interpreting FDG-PET/CT in the setting of lymphoma. Certain subtypes of B-cell lymphoma, particularly extranodal marginal zone lymphoma and peripheral T-cell lymphoma, display variable FDG uptake and can also be sometimes difficult to identify on contrast enhanced CT (CECT) [9]. In these cases, a baseline FDG-PET/CT is essential to facilitate meaningful interpretation of posttreatment imaging in terms of treatment response. A number of subtypes of lymphoma may be difficult or almost impossible to detect with FDG-PET/CT such as primary CNS lymphoma, testicular lymphoma, and gastric lymphoma. The normal brain and testes demonstrate intense FDG avidity and against this high background FDG activity, primary CNS and testicular lymphoma may be very difficult to detect without adjusting the image intensity thresholds [10]. Gastric lymphoma displays an extremely variable FDG uptake that may be difficult or impossible to differentiate from normal physiologic FDG uptake in the gastric mucosa [11]. Diffuse or heterogeneous bone marrow uptake of radiotracer in a pretreatment scan should not immediately suggest bone marrow involvement by lymphoma as this pattern of uptake can be seen in reactive myeloid hyperplasia [5, 12]. Focally increased FDG uptake can also be seen in the musculoskeletal system due to trauma, muscle activity, and degenerative change in synovial joints and also in brown fat which has a higher basal metabolic rate than adjacent tissues and may be mistaken for a lymphoma deposit. However, brown fat has a characteristic distribution and its detection is usually reasonably straightforward on FDG-PET/CT [13] (Figure 1). If patients with lymphoma are treated with granulocyte-colony stimulating factors, their bone marrow may show a diffuse increase in FDG uptake. It is very important to recognize the aforementioned pitfalls to avoid false-positive interpretation of FDG-PET/CT scans and hence potential overstaging of patients.

### 2.1. FDG-PET/CT Technique

There is a wide variance in the protocols used to obtain FDG-PET/CT images with variations in the use of intravenous contrast and the tube current (and hence radiation dose) used in the CT component of the study. In general, a patient who is undergoing an FDG-PET/CT scan will have their blood glucose level measured prior to the intravenous administration of FDG and if the serum glucose concentration is too high (above 140 mg/dL), a subcutaneous injection of rapid acting insulin may be considered [14]. The patient is placed in a recumbent position and instructed to limit movement as this will reduce muscle activity and prevent unwanted FDG uptake in skeletal muscle. An intravenous dose of FDG is administered (the dose is dependent on the patient's weight) and imaging is performed 1-2 hours (usually 1 hour) after injection as this allows a steady state of FDG uptake. Imaging is performed using an FDG-PET/CT scanner which integrates a multidetector CT scanner with a high-resolution PET scanner, facilitating the acquisition of three-dimensional images. Axial whole-body FDG-PET/CT images are acquired from the vertex or skull base to the upper thigh [8]. The “whole-body” nature of the imaging technique makes FDG-PET/CT ideal for evaluating systemic diseases such as lymphoma.

### 2.2. Standardized Uptake Value (SUV)

SUVs represent reproducible estimates of tissue glucose metabolic activity and can be used for semiquantitative analysis of findings on FDG-PET/CT in patients with lymphoma [15]. This is very useful in clinical practice as it allows accurate comparison of lesions on subsequent scans (i.e., to guide response to treatment) and can be used to evaluate the degree of residual metabolic activity in a treated mass. This helps physicians make clinical decisions regarding the use of salvage chemotherapy [16]. While the SUV is semiquantitative, a number of studies have demonstrated that visual assessment of treatment response in patients with lymphoma is adequate for determining whether an FDG-PET/CT scan is positive for disease [6, 7].

### 2.3. FDG-PET/CT versus CT

**Figure 2:**
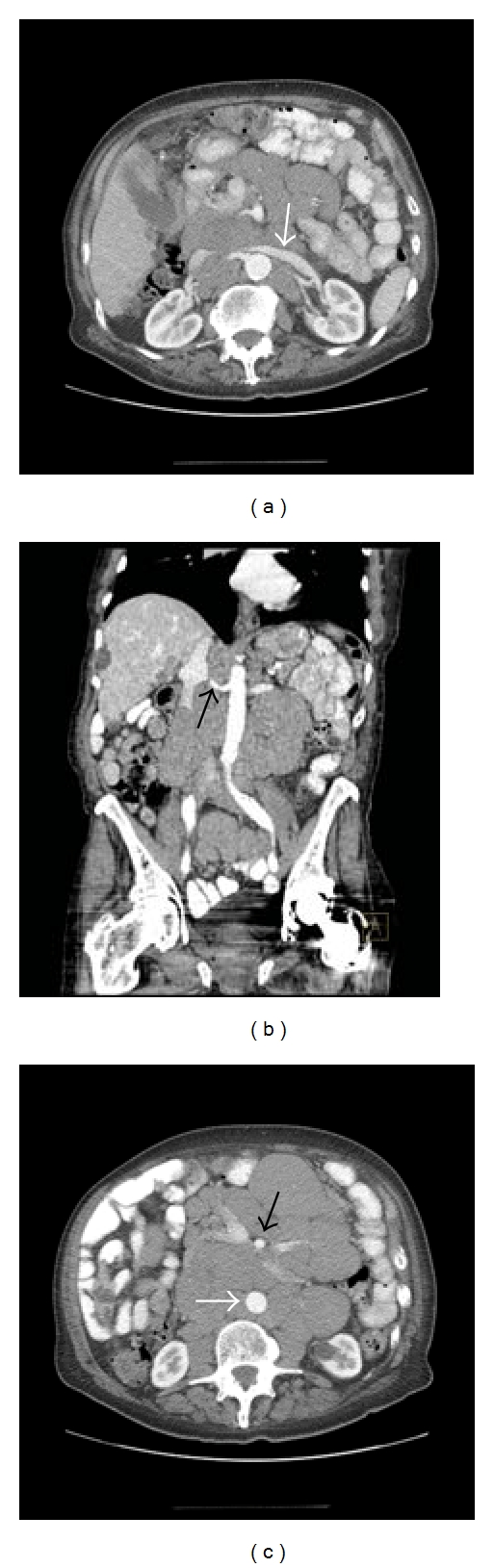
Axial (a) and coronal (b) contrast enhanced CT of the abdomen in a patient with chronic lymphocytic leukemia displaying encasement of vessels such as the left renal vein (white arrow) and right renal artery (black arrow) by lymph nodes masses, the so-called “sandwich sign.” Axial contrast enhanced CT (c) of the abdomen at a lower level in the same patient displaying elevation of the abdominal aorta off the vertebral column (white arrow) which is a characteristic feature of lymphoma and helps to differentiate lymphoma from other retroperitoneal masses, such as retroperitoneal fibrosis. Note the encasement of the superior mesenteric artery (SMA) by the lymph node mass and increased separation of the SMA from the aorta (black arrow).

FDG-PET/CT is often not the first-line imaging modality in the assessment of a patient with suspected lymphoma. CT is widely available and, in part due to ease of access and cost factors, it was the dominant radiologic tool for the assessment of lymphoma over the past three decades. Lymphoma displays homogenous attenuation on CT with characteristic patterns of disease such as vessel encasement and displacement (the so-called “sandwich sign” [17]) and spread across existing anatomic structures which indicates an aggressive, permeative disease process [8] (Figure 2). 

However, the fundamental limitation of CT in imaging lymphoma is that recognition of nodal involvement by disease is based solely on size criteria and the detection of bone marrow and extranodal involvement is limited. FDG-PET/CT incorporates a CT component in addition to a FDG-PET scan, and it has been demonstrated to have a higher sensitivity and specificity for the detection of nodal and extranodal sites of disease when compared to CT alone, thus improving baseline staging [8, 18–20]. It has long been recognized that CT is limited in its ability to assess the response of lymphoma to treatment as CT cannot differentiate between viable tumor and necrosis or fibrosis in patients presenting with persistent masses posttreatment [21]. The sensitivity and specificity of FDG-PET/CT has been shown to be superior to that of CT alone in the posttreatment assessment of lymphoma due to the addition of information regarding the metabolic activity of residual masses (RMs) detected on CT [8, 20].

### 2.4. FDG-PET/CT versus Gallium-67 Citrate Scintigraphy

The development and clinical utilization of the first molecular imaging agent for lymphoma assessment, namely, Gallium-67 citrate (^67^Ga), greatly improved the accuracy of treatment response assessment in patients with lymphoma [22–24]. ^67^Ga scanning is based on the accumulation of the isotope into viable lymphoma cells by binding to transferrin receptors [25]. It has been demonstrated that there is a significant difference in progression-free survival between patients with treated lymphoma that have a positive or negative ^67^Ga scan; however, the positive and negative predictive values of ^67^Ga imaging are suboptimal [22, 23]. There are a number of technical and clinical factors that have limited the use and acceptance of ^67^Ga imaging, namely, its low spatial resolution, low sensitivity and specificity, its limited use for imaging intra-abdominal disease (due to physiologic bowel uptake), and the time involved in performing the scan, which can take up to a week after ^67^Ga injection [25, 26]. ^67^Ga imaging has been largely replaced by FDG-PET, which is a radiolabelled isomer of glucose and is hence a very sensitive marker of glucose metabolism and metabolic activity [27]. FDG-PET is superior to ^67^Ga scintigraphy in terms of sensitivity for the detection of and specificity for malignant lymphoma [27–30]. The use of FDG-PET/CT tends to upstage patients due to the detection of additional sites of lymphomatous involvement such as the liver, spleen, and lung which can result in a change of treatment strategy [30–32].

## 3. FDG-PET/CT for Primary Staging of Aggressive B-Cell Lymphoma

**Figure 3:**
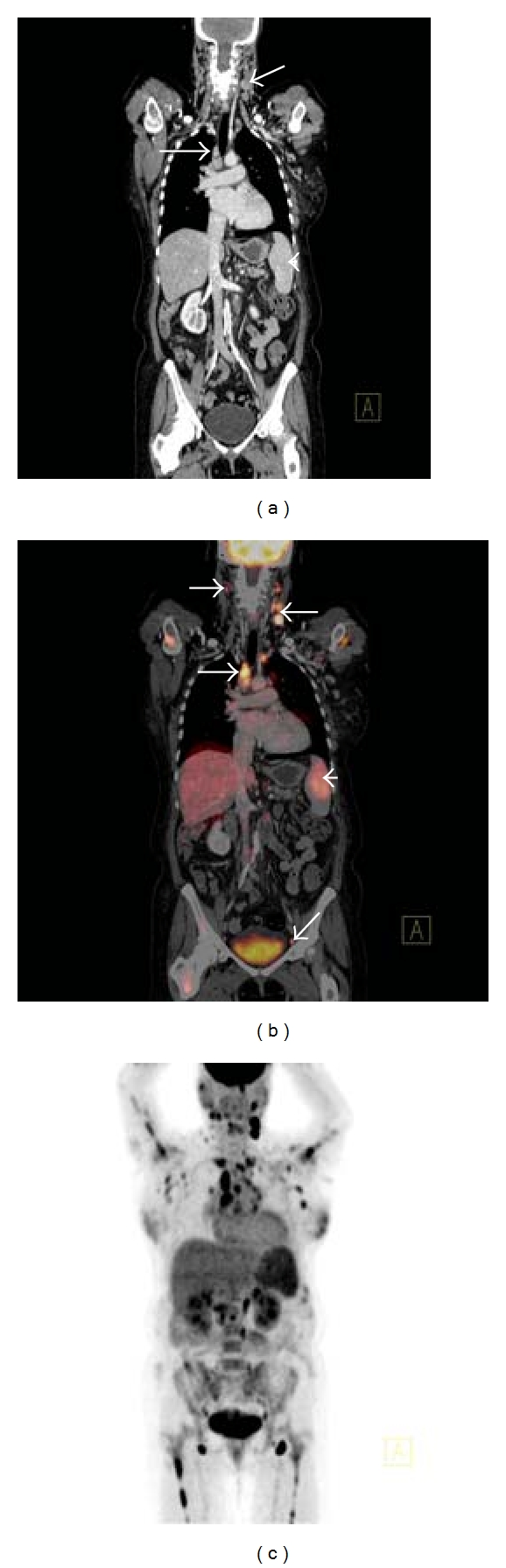
Coronal CT (a) demonstrating left cervical and right paratracheal lymphadenopathy (white arrows) and mild splenomegaly (white arrowhead) consistent with high-grade follicular lymphoma. Corresponding coronal fused FDG-PET/CT (b) demonstrates increased radiotracer uptake in the pathologically enlarged left cervical and right paratracheal lymphadenopathy (white arrows) and spleen (white arrowhead), but also abnormal FDG accumulation in right cervical and left external iliac lymph nodes (white arrows) that appeared normal by size criteria on the corresponding CT scan. Coronal PET MIP (c) in the same patient demonstrating widespread increased radiotracer uptake throughout the neck, thorax, abdomen, and proximal humeri and femora bilaterally; the widespread bone marrow involvement was not apparent on CT.

FDG-PET/CT has been demonstrated in multiple studies to be very sensitive for the detection of nodal and extranodal lymphoma at initial staging of patients prior to commencement of treatment [33–40]. In general, indolent FL is associated with low-grade FDG uptake, whereas more intense FDG accumulation is seen in more aggressive lymphomas, such as DLBCL [41]. In comparison to CT alone, FDG-PET/CT detects additional foci of FDG uptake, presumed to be lymphomatous deposits, in subcentimeter lymph nodes and particularly in extranodal sites such as the liver, spleen, bone marrow, and muscle in patients with DLBCL with an impact on disease stage (usually upstaging) in approximately 15–20% of patients and with an influence on clinical management in approximately 5–15% [33–37, 42]. FDG-PET/CT is more accurate for staging than both FDG-PET and CT alone, with equal sensitivity and better specificity [30]. Pretreatment FDG-PET/CT imaging acts as a baseline study to facilitate comparison to posttreatment scans to assess response to treatment and evaluate RMs for metabolic activity [6, 7]. FDG-PET/CT has been demonstrated to be effective in the detection of focal or multifocal bone marrow infiltration in patients with a negative iliac crest bone marrow biopsy (BMB) [43]. However, it is important to stress that FDG-PET/CT alone is not completely reliable in detecting limited bone marrow involvement by lymphoma, and, hence, it cannot entirely replace BMB in the initial diagnosis of lymphomas of any type as a negative FDG-PET/CT scan cannot definitively outrule involvement of the bone marrow by lymphoma [44] (Figure 3). 

Combined FDG-PET/CT imaging represents a functional and anatomical approach to lymphoma assessment which exploits the strengths of both modalities while at the same time minimizing the shortcomings of each individual modality. A number of studies have demonstrated that a staging FDG-PET/CT scan provides diagnostic information that is at least equal but likely superior to that which is provided by a separate FDG-PET and intravenous contrast enhanced CT (CECT) scan [7, 12]. FDG-PET provides the functional information lacking on CT images that may represent the only indication of disease (e.g., abnormal FDG uptake in a normal-sized lymph node on CT). CT provides superior anatomic detail and spatial resolution that is lacking in FDG-PET which enables better localization of FDG uptake and, importantly, may identify false-negative FDG-PET findings due to the limited spatial resolution of PET scanners or lack of FDG avidity of some subtypes of lymphoma. The anatomic correlation of FDG uptake that is afforded by FDG-PET/CT is also very useful in reducing the rate of false-positive findings due to physiologic FDG uptake in, for example, muscle or brown fat [45]. There is debate in the published literature regarding the benefits of using intravenous contrast during the CT portion of a FDG-PET/CT scan. Some authors suggest that the use of intravenous contrast reduces the number of indeterminate findings on FDG-PET/CT and results in the detection of a higher number of extranodal sites compared with FDG-PET/CT performed without intravenous contrast [46]. However, a number of studies suggest that an FDG-PET/CT scan performed without intravenous contrast and using a low radiation dose CT technique is an acceptable alternative to separate CECT and FDG-PET scans even when the known limitations of the unenhanced CT in the evaluation of the liver and spleen are taken into account [19, 47]. It is the opinion of the authors that, for initial staging of patients with lymphoma, an FDG-PET/CT scan using a standard radiation dose CT protocol and intravenous contrast is the optimal choice as a single imaging modality.

## 4. FDG-PET/CT for Restaging of Aggressive B-Cell Lymphoma

Restaging of lymphoma refers to imaging patients after completion of treatment or to determine the degree of known or suspected disease recurrence. This is distinct from treatment monitoring in which a patient is reimaged during treatment, usually after two to three cycles of a six-to-eight-cycle chemotherapy regimen [12]. The imaging evaluation of treatment response was previously limited to the assessment of changes in lymph node size with CT. This led to a common diagnostic dilemma regarding residual lymph node masses as to whether they represented posttreatment fibrosis or residual, viable malignancy. The presence of residual active disease is associated with early relapse and poor clinical outcome and usually necessitates further, more aggressive treatment [48, 49]. FDG-PET/CT is very useful in the restaging of lymphoma posttreatment as it can reliably discriminate between benign fibrosis (low or absent FDG uptake) and residual viable lymphoma (elevated FDG uptake) [5]. The International Harmonization Project (IHP) [6, 7] in lymphoma made a number of recommendations intended to reduce the rate of false-positive FDG-PET scan interpretation after completion of treatment.

1. FDG-PET/CT should not be performed for at least 3 weeks after chemotherapy and 8–12 weeks after radiotherapy to minimize confounding FDG uptake secondary to posttreatment inflammation.
2. RMs ≥ 2 cm in maximum transverse diameter should only be considered positive for residual disease on FDG-PET if their FDG avidity visually exceeds that of the mediastinal blood pool structures.
3. RMs with a maximum transverse diameter < 2 cm are considered FDG-PET positive if their FDG uptake is higher than the surrounding background tissue.
4. In the liver and spleen, focal lesions with FDG avidity greater than in surrounding liver or spleen parenchyma are considered positive for viable lymphoma.
5. Single or multiple foci of distinctly elevated FDG uptake in the bone marrow is positive for residual disease.
6. Visual assessment alone is adequate for determining whether FDG-PET scans are positive or negative at the conclusion of treatment and SUV measurement is not necessary.

**Figure 4:**
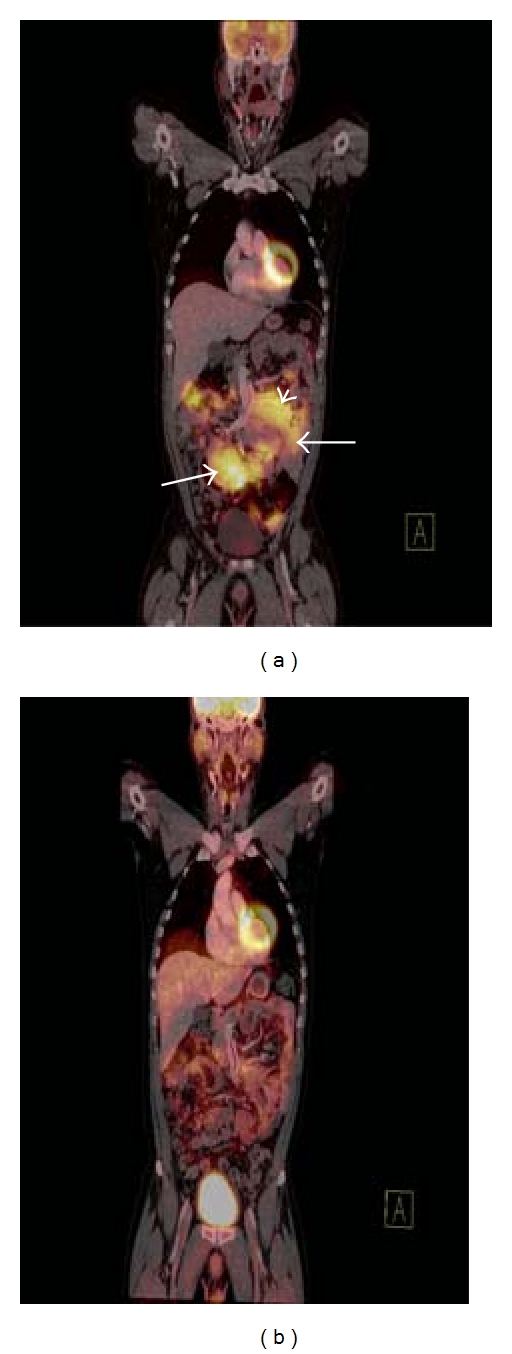
Coronal fused FDG-PET/CT image (a) demonstrating increased radiotracer uptake in multiple intra-abdominal lymph node masses in a patient with DLBCL (white arrows). Note how a large soft tissue mass displaces the small bowel and the mesenteric vessels (white arrowhead). Coronal fused FDG-PET/CT image (b) in the same patient demonstrating complete resolution of the previously described soft tissue masses. The observed residual FDG avidity is within bowel and is normal. This FDG-PET/CT confirms complete response to treatment.

The use of FDG-PET/CT in restaging lymphoma is supported by a large body of evidence that shows that FDG-PET/CT has a high negative predictive value (NPV) that exceeds 80% in practically all reported studies for aggressive NHL, with the reported 10–20% false-negative rate mainly attributed to the inability of FDG-PET/CT to detect microscopic disease which results in future relapse [6, 7, 12, 50–53] (Figure 4). However, the positive predictive value (PPV) of FDG-PET/CT is reported in the moderate range of 70–80% which is significantly lower and more variable than its NPV and is due to the recognized false-positive rate on FDG-PET due to persistent metabolic activity in RMs posttreatment that often represents inflammatory change rather than residual disease. The PPV of FDG-PET/CT is substantially higher than that of CT alone which has a reported PPV in the range of 40–50% in posttreatment aggressive NHL leading to a considerably higher accuracy of FDG-PET/CT for response assessment when compared to CT alone [6, 7, 12, 50–53]. The significantly higher accuracy of FDG-PET/CT for response assessment when compared to CT alone justifies the increased cost associated with dual modality imaging when compared to CT or PET alone [54, 55].

## 5. FDG-PET/CT for Interim Treatment Monitoring

The use of FDG-PET/CT for treatment monitoring is based on the assumption that interim imaging, typically performed after two to three cycles of chemotherapy, provides accurate prediction of response to current treatment and ultimate patient outcome [30, 56]. The application of FDG-PET/CT in interim treatment monitoring of lymphoma has been highly successful in Hodgkin's disease [57, 58]. However, the benefits of FDG-PET/CT in the management of aggressive NHL are less clear cut with conflicting reports in the literature regarding the usefulness of interim FDG-PET/CT in predicting treatment response and patient outcome with most reports recommending further prospective studies to ascertain the role of FDG-PET/CT in this respect [59–61]. There are a number of reported studies in the literature that support this premise, particularly with regard to the high NPV of interim FDG-PET/CT in patients with aggressive NHL [62–66]. It is interesting to note that the PPV and, hence, accuracy of interim FDG-PET scans that are visually interpreted (i.e., without the use of semiquantitative SUV) appear to depend on the “strictness” of criteria used to define a scan as positive with a number of studies demonstrating that a more “liberal” interpretation of FDG-PET findings at the site of RMs on interim FDG-PET results in higher PPV and accuracy with no compromise in NPV [66–68]. This “liberal” visual interpretation involves considering an FDG-PET scan as negative if it displays only minimal FDG uptake that is similar or only slightly greater in intensity than FDG uptake in normal liver [66–68]. If these “liberal” criteria for defining FDG-PET negativity are used, interim FDG-PET was found to be at least as accurate for predicting patient outcome as end of treatment FDG-PET interpreted using “strict” criteria that does not allow any residual FDG uptake in RMs [68].

**Figure 5:**
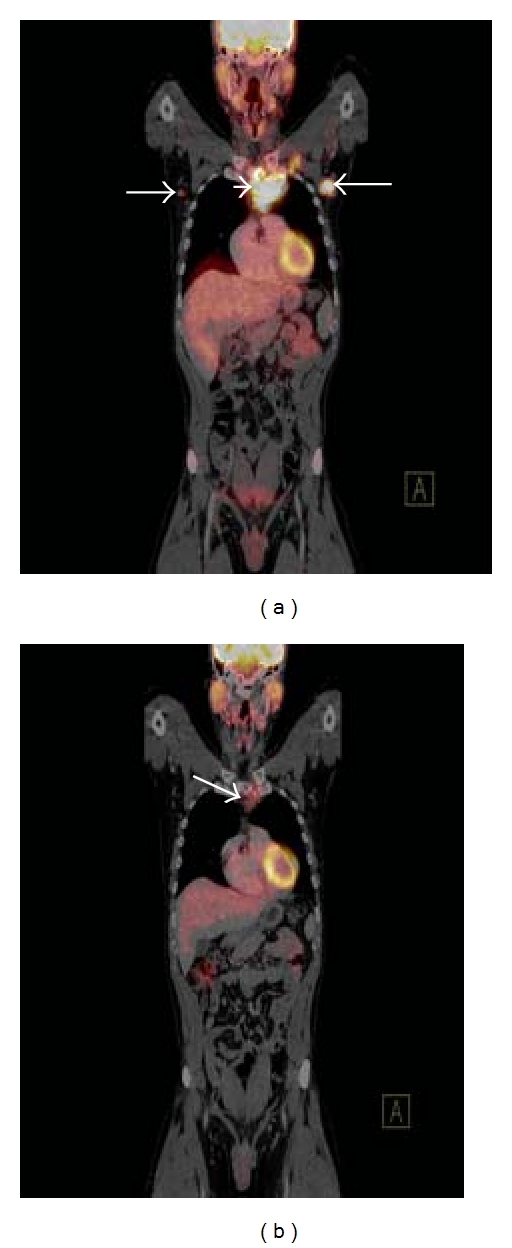
Coronal fused FDG-PET/CT image (a) of a patient with nodular sclerosis type Hodgkin's lymphoma prechemotherapy demonstrating an FDG-avid lymph node mass in the superior mediastinum (white arrowhead) and hypermetabolic bilateral axillary lymphadenopathy (white arrows). Interim staging coronal fused FDG-PET/CT image (b) in the same patient after 3 cycles of chemotherapy demonstrating a significant interval decrease in both the degree of FDG uptake and the size of the lymph node mass in the superior mediastinum (white arrow) with resolution of the axillary lymphadenopathy.

Thus, the prognostic power of an interim FDG-PET/CT scan is significant with a number of studies indicating that a negative interim FDG-PET scan in patients with aggressive NHL confers an excellent prognosis while a positive study identifies patients who are likely to experience early relapse and in whom a change in treatment regimen may be beneficial [51, 65, 66]. It must be stressed, however, that interim FDG-PET/CT has a relatively limited PPV when compared to its NPV due to the occurrence of false-positive results due to posttreatment inflammation in RMs. Thus, caution should be exercised when considering changing a patient's treatment regimen based on the findings of FDG-PET/CT imaging alone and it is reasonable to biopsy this patient group if such a change in treatment is being contemplated [69]. Interim FDG-PET/CT scans appear to be very useful in providing a basis for risk-adjusted or “personalized” therapy, whereby the results of interim FDG-PET/CT scans will identify patients who have responded well to initial treatment (i.e., they have a negative interim scan) and therefore will be suitable for a more limited treatment regimen (i.e., reduced numbers of chemotherapy cycles with less treatment-related toxicity) without compromising treatment efficacy or those patients in whom response to initial treatment was poor (i.e., they have a positive interim scan) and therefore an early change of treatment regimen may improve patient outcome [69] (Figure 5). 

## 6. FDG-PET/CT for Posttreatment Surveillance

The use of FDG-PET/CT for posttreatment surveillance in patients with aggressive NHL is performed following completion of treatment and in the absence of clinical, biochemical, or radiologic evidence of disease recurrence (i.e., a complete response (CR) to treatment) with the aim of early detection of disease relapse and hence earlier treatment to improve patient outcome [25]. A recent study reported positive results with the use of surveillance FDG-PET scanning in 421 lymphoma patients (160 HL, 183 aggressive NHL, and 78 indolent NHL) who were imaged at 6-month intervals for 2 years and then yearly thereafter following initial CR. This study demonstrated that the detection rate of proven relapses was higher with FDG-PET compared with CT alone or clinical assessment in aggressive NHL (31% versus 25% and 22%, resp.) and indolent NHL (60% versus 49% and 38%, resp.) [70]. The same study identified 36 patients with borderline positive FDG-PET findings who subsequently underwent biopsy that demonstrated lymphoma relapse in 24 patients and 12 false-positive findings (nine lymph nodes displaying reactive hyperplasia and three patients with sarcoid-like granulomatosis) [70]. While this study is encouraging, it did not determine whether surveillance FDG-PET scanning was cost effective and whether the results of surveillance imaging improved clinical outcomes. A study by Petrausch et al. [71] demonstrated that FDG-PET/CT detected recurrent DLBCL after first CR with a high PPV (85%); however, the authors report that it should not be used routinely and only in selected high-risk patients (such as patients >60 years of age and patients <60 years of age with clinical signs of relapse) to reduce radiation dose to patients in remission and costs. FDG-PET/CT has failed to demonstrate a clear benefit in posttreatment surveillance, and thus, until large prospective trials address these issues, the use of FDG-PET/CT for disease surveillance will remain controversial with some authors suggesting that its use should be limited to a clinical trial setting [69, 72]. If there is a low clinical suspicion for recurrence of patients with treated initially FDG-avid lymphomas, then FDG-PET/CT scan using a low radiation dose CT protocol without intravenous contrast may be a reasonable choice of a single lower-dose imaging modality for follow-up assessment. The routine use of FDG-PET/CT in disease surveillance is not yet supported by a sufficient body of evidence to justify its cost and associated radiation dose, and there are also some issues in relation to false-positive FDG-PET/CT studies during disease surveillance which could cause unwarranted patient distress and further add to the cost of management.

## 7. FDG-PET/CT for Assessment of Transformation

The transformation of indolent lymphoma to a higher grade of lymphoma can occur, most commonly with DLBCL, and is associated with a very poor prognosis and is an indication for aggressive, high-dose chemotherapy and postremission stem cell transplantation [73, 74]. FDG-PET/CT is useful to confirm the clinical suspicion of transformation of an indolent lymphoma to a more aggressive histology by identifying sites of abnormally high FDG uptake (indolent lymphoma normally has low-grade FDG uptake) and also guiding selection of an optimal biopsy site for pathologic confirmation of transformation [41, 73]. Due to the significant overlap in the degree of FDG uptake between indolent and aggressive lymphomas, FDG-PET/CT cannot entirely replace biopsy in the assessment of transformation of lymphoma but rather may be a useful alternative when a biopsy is not practical due to technical or clinical reasons [41, 75].

## 8. Conclusion

FDG-PET/CT is an established imaging modality that has been proven to be of benefit in the management of aggressive B-cell NHL, such as DLBCL and advanced stage FL. The combination of anatomic and functional imaging afforded by FDG-PET/CT has led to superior sensitivity and specificity in the primary staging, restaging, and assessment of response to treatment of hematological malignancies when compared to FDG-PET and CT alone. The use of FDG-PET/CT for posttreatment surveillance imaging remains controversial, and further study is needed to ascertain whether this modality is cost effective and appropriate for use in this setting.
